# Coadministration of bedaquiline and pyrifazimine reduce exposure to toxic metabolite N-desmethyl bedaquiline

**DOI:** 10.3389/fphar.2023.1154780

**Published:** 2023-10-04

**Authors:** Yangming Ding, Haiting Liu, Furun Wang, Lei Fu, Hui Zhu, Shuang Fu, Ning Wang, Xiaomei Zhuang, Yu Lu

**Affiliations:** ^1^ Department of Pharmacology, Beijing Key Laboratory of Drug Resistance Tuberculosis Research, Beijing Tuberculosis and Thoracic Tumor Research Institute, Beijing Chest Hospital, Capital Medical University, Beijing, China; ^2^ State Key Laboratory of Toxicology and Medical Countermeasures, Beijing Institute of Pharmacology and Toxicology, Beijing, China

**Keywords:** bedaquiline, drug-drug interaction, N-desmethyl-bedaquiline, pyrifazimine, tuberculosis

## Abstract

**Background:** A new, effective anti-tuberculosis (TB) regimen containing bedaquiline (BDQ) and pyrifazimine (TBI-166) has been recommended for a phase IIb clinical trial. Preclinical drug–drug interaction (DDI) studies of the combination of BDQ and TBI-166 have been designed to support future clinical trials. In this study, we investigated whether a DDI between BDQ and TBI-166 affects the pharmacokinetics of BDQ.

**Methods:** We performed *in vitro* quantification of the fractional contributions of the fraction of drug metabolism by individual CYP enzymes (*f*_m_) of BDQ and the inhibition potency of key metabolic pathways of TBI-166. Furthermore, we conducted an *in vivo* steady-state pharmacokinetics study in a murine TB model and healthy BALB/c mice.

**Results:** The *in vitro f*_m_ value indicated that the CYP3A4 pathway contributed more than 75% to BDQ metabolism to N-desmethyl-bedaquiline (M2), and TBI-166 was a moderate (IC_50_ 2.65 µM) potential CYP3A4 inhibitor. Coadministration of BDQ and TBI-166 greatly reduced exposure to metabolite M2 (AUC_0-t_ 76310 vs 115704 h ng/mL, 66% of BDQ alone), whereas the exposure to BDQ and TBI-166 did not changed. The same trend was observed both in healthy and TB model mice. The plasma concentration of M2 decreased significantly after coadministration of BDQ and TBI-166 and decreased further during treatment in the TB model.

**Conclusions:** In conclusion, our results showed that the combination of BDQ and TBI-166 significantly reduced exposure to the toxic metabolite M2 by inhibiting the activity of the CYP3A4 pathway. The potential safety and efficacy benefits demonstrated by the TB treatment highly suggest that coadministration of BDQ and TBI-166 should be studied further.

## 1 Introduction

The World Health Organization (WHO) estimates that in 2021, 1.6 million people died from tuberculosis (TB) and more than 10 million people have suffered from the disease worldwide, with nearly 160,000 cases of drug-resistant tuberculosis (DR-TB). The treatment of DR-TB usually requires a combination of new anti-TB drugs (WHO, 2022).

Bedaquiline (BDQ) is the first new drug in the last 70 years for the treatment of DR-TB and is considered the most effective drug for the treatment of DR-TB today, which was officially approved for clinical use by the U.S. Food and Drug Administration in 2012. Since 2018, it has been listed by the WHO as a Group A drug in the DR-TB treatment guidelines and as a core drug in regimens for treating DR-TB (Li Y. et al., 2019). However, BDQ induces phospholipidosis and has been associated with QT prolongation. Both *in vitro* and clinical studies suggest that N-desmethyl-bedaquiline (M2) is a more potent inducer of phospholipidosis than the parent drug and it is thought to be responsible for the QT prolongation associated with BDQ use (Ngwalero et al., 2021).

Pyrifazimine (TBI-166) is an anti-TB drug candidate obtained by optimizing the riminophenazine drug, clofazimine (Lu et al., 2011). Previous studies on murine model have shown that TBI-166 has a strong anti-TB effect and the synergistic effects of TBI-166 and BDQ significantly increased anti-TB activity by more than 50% compared with BDQ treatment group (Zhang et al., 2019). Some typical regimens that contain BDQ and TBI-166 as the partner agent have shown stronger bactericidal and sterilizing activity than the first-line HRZE regimen. Therefore, BDQ and TBI-166 are a promising combination for treating DR-TB and have been recommended for further study in a phase IIb clinical trial (Ding et al., 2022; Liu et al., 2023).

However, the synergistic effects of TBI-166 and BDQ require their possible drug–drug interactions (DDIs) to be investigated. Drug-drug interactions (DDIs) involving drug-metabolizing enzymes, such as the cytochrome P450 (CYP) family enzymes, are the most common types of DDIs encountered in clinical practice (Ye et al., 2021). BDQ, which is primarily metabolized by CYPs and exhibits high plasma protein binding, poses a significant risk of DDIs when combined with other drugs (Van Heeswijk et al., 2014; Pandie et al., 2016a). Given that the lead riminophenazine drug, clofazimine, is a moderate-to-strong CYP3A4/5 inhibitor (Horita and Doi, 2014; Sangana et al., 2018), riminophenazine drug TBI-166 also has the potential for DDIs with BDQ that should be investigated in the preclinical phase.

In the current study, quantitative fractional contributions of a panel of the fraction of drug metabolism by individual CYP enzymes (*f*_m_) of the victim drug, BDQ, and the strength of the inhibition of key metabolic pathways for the DDI of the perpetrator drug, TBI-166, were assayed *in vitro*. We then investigated whether DDIs during the coadministration of BDQ and TBI-166 affect the pharmacokinetics of BDQ in healthy mice and in a murine model of TB *in vivo*.

## 2 Method

### 2.1 Chemicals and materials

BDQ and propranolol were purchased from Sigma-Aldrich (St. Louis, MO, United States); TBI-166 was provided by the Institute of Materia Medica, Peking Union Medical College and Chinese Academy of Medical Sciences (Beijing, China); N-desmethyl-bedaquilin (M2) were purchased from Toronto Research Chemicals Inc. (Toronto, Canada); Probe substrates and associated metabolites for CYPs were purchased from BD Bioscience (San Jose, CA, United States); β-nicotinamide adenine dinucleotide phosphate (NADPH) was purchased from Roche (Roche, Switzerland); dimethyl sulfoxide, formic acid, acetic acid were purchased from Sigma-Aldrich (St. Louis, MO, United States);, acetonitrile, and methanol were purchased from Fisher Co. Ltd. (Waltham, MA, United States); Human liver microsomes (HLM; 150-donor pool, mixed sex) and recombinant human cytochrome P450s (rCYPs; rCYP1A2, 2A6, 2B6, 2C8, 2C9, 2C19, 2D6, 2E1, 2J2, 3A4, 3A5, and 4F2) were purchased from BD Gentest (Woburn, MA, United States); Milli-Q (Millford, MA, United States) water was used throughout the study.

### 2.2 Animals

#### 2.2.1 Pharmacokinetic interaction of the combination of BDQ and TBI-166 in BALB/c mice

72 male BALB/c mice, aged 6 weeks and weighing 18–20 g, were purchased from the Beijing Vital River Laboratory Animal Technology Company. The mice were placed in cages with three mice per cage and given a week to adjust to their surroundings before the experiment. The housing was kept at a constant temperature and humidity using an air conditioning system.

#### 2.2.2 Interaction of BDQ and TBI-166 in murine model of tuberculosis

30 female BALB/c mice, aged 6 weeks and weighing 18–20 g, were purchased from the Beijing Vital River Laboratory Animal Technology Company. The mice were placed in cages with five mice per cage and given to 1 week to adjust to their surroundings before the experiment. The housing was kept at a constant temperature and humidity using an air conditioning system. Murine TB model were aerosol infected with mouse-passaged *M. tuberculosis* H37Rv by using an inhalation exposure system (099C A4224, Glas-Col). The experiment was started 4 weeks after the infection.

### 2.3 Reaction phenotyping of BDQ in recombinant P450 enzymes and HLM

#### 2.3.1 Incubations of BDQ in rCYPs

A stock solution of BDQ was prepared in DMSO. rCYPs (rCYP1A2, 2A6, 2B6, 2C8, 2C9, 2C19, 2D6, 2E1, 2J2, 3A4, 3A5, and 4F2) were co-incubated with BDQ to investigate its metabolizing enzyme phenotyping. The mixture containing BDQ (1 μM), phosphate-buffered saline (PBS; 100 mM, containing MgCl_2_ 3 mM, pH 7.4), rCYPs (20 pM/mL), and NADPH (1 mM) had a final volume of 250 μL. Each incubation was performed in triplicate. Incubation without NADPH was used as the negative control. After incubation at 37°C for 1 h, the reaction was terminated by the addition of acetonitrile (containing IS, propranolol 100 ng/mL) at predetermined time points (0, 5, 10, 30, and 60 min). After the mixture was vortexed, all samples were centrifuged at 4000 g for 20 min. Next, 5 μL solution was injected into a liquid chromatography–tandem mass spectrometry (LC-MS/MS) system for analysis (Xu et al., 2018).

#### 2.3.2 Incubations of BDQ in HLM with specific inhibitors to CYP isoforms

HLM inhibition studies were performed using isoform selective chemical inhibitors sulfaphenazole (CYP2C9), montelukast (CYP2C8), nootkatone (CYP2C19), ketoconazole (3A4) and positive control (without chemical inhibitors) were co-incubated in HLM with BDQ to examine BDQ reaction phenotyping. Reaction mixtures were prepared containing selective chemical inhibitors, PBS (100 mM, containing MgCl_2_ 3 mM, pH 7.4), HLM (0.5 mg/mL), and BDQ (1 μM). The mixture was preincubated at 37°C for 5 min. Next, reactions were initiated by the addition of NADPH (1 mM) and terminated by addition of acetonitrile (containing IS, propranolol 100 ng/mL) at predetermined time points (0, 5, 10, 30, and 60 min). After the mixture was vortexed, all samples were centrifuged at 4000 g for 20 min. 5 μL solution was injected into a LC-MS/MS system for analysis (Lindmark et al., 2018).

### 2.4 Determination of IC_50_ of TBI-166

The direct inhibition potential of TBI-166 against P450 was determined using the human marker probe reactions for P450 enzymes in human liver microsomes. Briefly, substrate concentrations in the incubation mixture were selected to be around their K_m_. Eight different concentrations of TBI-166 (from 0.05 to 10 μM) were incubated in human liver microsomes respectively. Incubations were carried out at 37°C in a shaking water bath. The incubation mixtures contained HLM (0.5 mg/mL), probe substrates in the presence or absence of TBI-166 in PBS (100 mM, containing MgCl_2_ 3 mM, pH 7.4). The reactions were initiated by adding NADPH (1 mM) after preincubated the above mixtures at 37°C for 5 min. Positive controls without TBI-166 were included simultaneously. Stock solutions of the test drugs were prepared in acetonitrile. All measurements were performed in triplicate. All incubations were stopped at 10 min by adding acetonitrile (containing IS, propranolol 100 ng/mL). Protein was removed by centrifugation at 4,000 *g* for 20 min and analyzed by LC-MS/MS (Li Z. et al., 2019).

### 2.5 Pharmacokinetics of BDQ in the presence or absence of TBI-166

#### 2.5.1 Pharmacokinetic interaction of the combination of BDQ and TBI-166 in BALB/c mice

All mice were randomly divided into three groups, single administration of BDQ at 25 mg/kg or single administration of TBI-166 at 20 mg/kg or co-administration of BDQ at 25 mg/kg and TBI-166 at 20 mg/kg were administered as QD dosing by gavage for consecutive 21 days. Blood and tissue were collected from 3 mice per group per time point, at 0, 0.5, 1, 2, 3, 8, 24, 48, 72, and 168 h after last dose of BDQ, TBI-166 or coadministration of BDQ and TBI-166. Blood was harvested from veins into tubes and centrifuged within 1 h after collection. Plasma was obtained and stored at −20°C for bioanalysis. Lung or spleen samples were homogenized with 3 mL cold methanol (4°C). The homogenates were precipitated with acetonitrile (containing IS) and analyzed.

#### 2.5.2 Interaction of BDQ and TBI-166 in murine model of tuberculosis

All mice were randomly divided into three groups, multiple doses of single administration of TBI-166 at 20 mg/kg or single administration of BDQ at 25 mg/kg or coadministration of BDQ 25 mg/kg and TBI-166 20 mg/kg were administered for 4 weeks or 8 weeks. Five mice from each group were sacrificed after 4 or 8 weeks of treatment to assess the plasma concentration at 3 hours post-dosing. Blood was collected from veins into tubes and centrifuged within 1 h after collection. Plasma was harvested and stored at −20°C for bioanalysis.

### 2.6 Sample preparation

A 50 μL aliquot of mice plasma was spiked with 450 μL of acetonitrile (containing IS, propranolol 100 ng/mL) and subsequently vortex-mixed for 5 min for protein precipitation. After centrifugation of the samples at 12,000 g for 10 min, 100 µL of the supernatant was transferred, and 5 µL of the resulting sample solution was injected into an LC-MS/MS system for determination of BDQ, TBI-166, and M2.

### 2.7 Bioanalysis method

BDQ, TBI-166 and M2 were determined using a LC-MS/MS system consisting of an Applied Biosystem ultraLC110 HPLC instrument (Foster City, CA, United States) and Applied Biosystem 4000 Q-Trap (Foster City, CA, United States) equipped with an electrospray ionization interface.

The CYPs probe substrates were determined using a LC-MS/MS system consisting of a Shimadzu LC-20AD HPLC instrument (Tokyo, Japan) and Applied Biosystem 5,000 triple quadrupole mass spectrometer detector (Foster City, CA, United States) equipped with an electrospray ionization interface.

#### 2.7.1 Analysis of BDQ, M2 and TBI-166

The chromatographic separation was performed on a Zorbax Eclipse Plus C18 column (2.1 mm × 50 mm, i. d, 3.5 µM particle size; Agilent, United States) maintained at 40°C. The mobile phase consisted of 0.1% formic acid in water (A) and 0.1% formic acid acetonitrile (B); it was run according to the following gradient programs at a flow rate of 0.4 mL/min: 90% A (0–1 min), 90%–10% A (1.1–1.5 min), 10% A (1.5–3.5 min), 90%–10% A (3.5–4.0 min) and 90% A (4.0–5.0 min) for the BDQ, M2 and TBI-166 assays. The mass detector with electrospray ionization interface was operated in positive ion mode and set according to the following conditions: spray voltage, 5,500 V; spray temperature, 450°C; curtain gas, 20 psi; source gas 1, 60 psi; and source gas 2, 60 psi. The quantification was performed through MRM of the molecular ion to the related product ion for each compound. The MRM transitions of BDQ, M2, TBI-166, and propranolol were m/z 555.3→58.1, 537.2→310.1, 590.1→478.1, and 260.1→116.1 respectively. The peak area ratio of the analyzed BDQ, TBI-166 and M2 versus IS was used for calculating the concentration.

#### 2.7.2 Analysis of CYP probe substrate metabolites

Metabolites of probe substrates were separated using a Gemini C18 column (2.1 × 50 mm, i. d, 3.5 µM particle size; United States). A LC gradient consisting of 0.1% formic acid aqueous solution (v/v, mobile phase A) and 0.1% formic acid in acetonitrile (v/v, mobile phase B) at a flow rate of 0.6 mL/min in a 5 min run. The LC separation program was as follows: 0–0.5 min, 2% B; 0.5–3.5 min, from 2% to 90% B; 3.5–4.0 min, kept at 90% B; 4.0–5.0 min, 2% B. A 10 μL sample was injected for analysis. The analytes and internal standard were detected by positive ion spray in the multiple-reaction-monitoring modes (MRM).

### 2.8 Data analysis

The *in vitro* f_m_ value of each CYPs in individually expressed P450 enzymes incubated by intrinsic clearance (CL_int(rP450j)_, mL/min/mg protein) of CYPs was calculated was calculated as Eqs 1–3. CL_int(rP450j)_ was determined based on metabolite generation, and the percent contribution of each P450 isozyme (f_m_, CYP) was calculated based on the CL_int_ of individual CYP450s (CL_int P450j_).

$${\text{CL}}_{\mathrm{int}\left(\text{rP}450j\right)}=V_{\max}/K_{m}$$ (1)

$${\text{CL}}_{\mathrm{int}\;P450j}={\text{CL}}_{\mathrm{int}\;\left(\mathrm{rP}450j\right)}\times {P450}_{j}\text{abundance}$$ (2)

$$f_{m},\text{CYP}=\frac{{\text{CL}}_{\mathrm{int}\;P450j}}{\sum_{j=1}^{n}\left({\text{CL}}_{\mathrm{int}\;P450}\right)}\times 100$$ (3)

(Chen et al., 2011; Achour et al., 2014)

The *in vitro* f_m_ value of each CYP as chemical inhibitors (f_m_,CYP_i_) of defined selectivity in HLM incubation by intrinsic clearance (Cl_int_, mL/min/mg protein) was calculated as Eq. 4:

$$f_{m},{\text{CYP}}_{i}=\frac{{\text{CL}}_{\mathrm{int}}\text{without}\;\text{inhibitor}-{\text{CL}}_{\mathrm{int}}\text{with}\;\text{inhibitor}}{{\text{CL}}_{\mathrm{int}}\text{without}\;\text{inhibitor}}\times 100$$ (4)

(Lindmark et al., 2018)

IC_50_ values were calculated using nonlinear regression analysis with GraphPad Prism 7 (GraphPad Software Inc., San Diego, CA). The following pharmacokinetic parameters were estimated from the plasma concentration-time curve for all mice using a non-compartmental model for sparse sampling data of the WinNonlin software (Pharsight, Mountain View, CA, United States): peak plasma concentration (C_max_); time (T_max_); terminal elimination half-life (t_1/2_); area under the plasma concentration-time curve (AUC) from zero to the last sample collection time (AUC_0-t_); AUC from zero to infinity (AUC_0-∞_); and mean residence time (MRT). All data are expressed as the mean ± SD. Plasma concentrations of BDQ, M2 and TBI-166 in murine model of tuberculosis between different groups were analyzed by one-way analysis of variance with Dunnett’s *post hoc* test to correct for multiple comparisons. The significance level was 0.05. SPSS (19.0, version for Windows, SPSS) was used for all statistical analyses.

## 3 Results

### 3.1 Role of CYPs in BDQ metabolism

**FIGURE 1:**
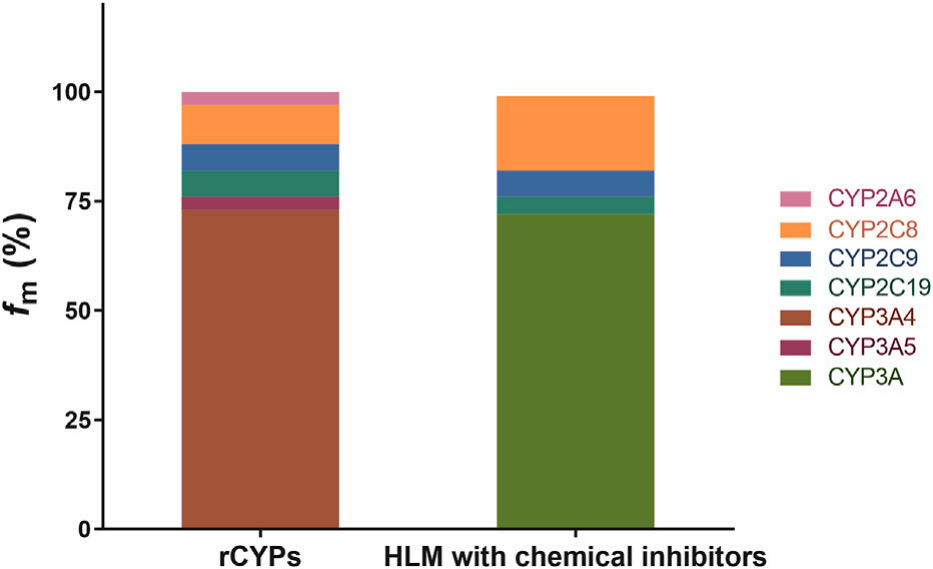
Role of CYPs in BDQ metabolism. The detailed incubation conditions are presented in the experimental procedures (*n* = 3); BDQ and M2 were determined by HPLC-MS/MS. *f*_m_ values for BDQ using the rCYPs method are as follows: CYP2A6 (3.1%), CYP2C8 (8.8%), CYP2C9 (5.8%), CYP2C19 (5.7%), CYP3A4 (73.1%), and CYP3A5 (3.5%); *f*_m_ values for BDQ using the HLM with chemical inhibitors are as follows: CYP2C8 (17.1%), CYP2C9 (6.2%), CYP2C19 (3.9%) and CYP3A4 (72.8%). *f*_m_, the fraction of drug metabolism by individual CYP enzymes; rCYPs, recombinant human cytochrome method P450s; HLM; human liver microsomes with chemical inhibitor method.

M2 is the major circulating metabolite of BDQ (Smyej et al., 2017). *In vitro* methods of estimating the relative contributions, *f*_m_, of specific human CYPs to the overall metabolism of BDQ were investigated and verified by two standard methods (Doran et al., 2022): by individually expressed CYPs and by chemical inhibitors with defined selectivity in HLMs. The calculated *f*_m_ of the CYPs involved in BDQ metabolism are shown in Figure 1.

The metabolic phenotyping of BDQ was performed for 12 individual CYPs by using HPLC-MS/MS. The formation of M2 during the enzyme-catalyzed reaction suggests that this pathway contributes to BDQ metabolism (Liu et al., 2014), The summary *f*_m_ value was set as 100% for the total clearance of BDQ. We found that of the 12 tested CYPs, six were involved in BDQ metabolism (CYP2A6, CYP2C8, CYP2C9, CYP2C19, CYP3A4, and CYP3A5) as shown in the figure. The remaining six CYPs were not involved in BDQ metabolism. The metabolic rates for the expressed CYPs were calculated by Eqs. 1–4 and the results are shown in Figure 1 as the “rCYPs” column. The *f*_m_ values of CYP3A4 and CYP3A5 were calculated as 73.1% and 3.5%, respectively, and the CYP3A family, which contains CYP3A4 and CYP3A5, was responsible for more than 75% of BDQ clearance and M2 generation. Thus, CYP3A is a major pathway for BDQ clearance. CYP2A6, CYP2C8, CYP2C9, and CYP2C19 were also involved in BDQ N-demethylation, with *f*_m_ values for BDQ of 3.1%, 8.8%, 5.8%, and 5.7%, respectively. The “HLM with chemical inhibitors” column in Figure 1 shows the contribution of each pathway calculated by chemical inhibitors with defined CYP selectivity in HLMs. These results indicated a contribution of nearly 75% to BDQ clearance by the CYP3A family, also demonstrating that this is a major BDQ clearance pathway. The *f*_m_ values for BDQ clearance obtained by selective chemical inhibitors in HLMs for CYP2C8, CYP2C9, and CYP2C19 were 17.1%, 6.2%, and 3.9%, respectively.

### 3.2 Inhibitory effect of TBI-166 on CYP-selective probe substrate metabolism

To further investigate the inhibitory effect of TBI-166 on the key pathway of BDQ metabolism, the comparative inhibitions of major CYP isoforms (CYP1A2, CYP2A6, CYP2B6, CYP2C8, CYP2C9, CYP2C19, CYP2D6, CYP2J2, CYP2E1, and CYP3A4) were determined for TBI-166 in HLMs by using marker probe substrates. The inhibitory effect (IC_50_) of TBI-166 on the activity of human CYPs is shown in Table 1.

**TABLE 1:** Inhibitory effects of TBI-166 on human CYP activity determined by *in vitro* screening.

	*C*_max_ (μg/mL), p.o. dosage^a^	Inhibition, IC_50_ (μM)^a^
CYP1A2	0.70 (1.19 μM, C_max_ observed at human equivalent dose of 20 mg)	0.55
CYP2A6		1.38
CYP2B6		4.35
CYP2C8		>50
CYP2C9		5.45
CYP2C19		4.49
CYP2D6		1.60
CYP2J2		>50
CYP2E1		0.93
CYP3A4 MDZ		17.36
CYP3A4 TEST		2.65

^a^ Positive controls: for CYP1A2, α-naphthoflavone (IC_50_), 0.33 µM; for CYP2C9, sulfaphenazole (IC_50_), 0.40 µM; for CYP2C19, nootkatone (IC_50_), 48.41 µM; for CYP2D6, quinidine (IC_50_), 0.017 µM; for CYP3A4 (MDZ), ketoconazole (IC_50_), 0.18 µM; CYP3A4 (TEST), ketoconazole (IC_50_), 0.45 µM. MDZ: midazolam; TEST: testosterone.

The role of CYPs in BDQ metabolism indicated that inhibitors of CYP2C8, CYP2C9, CYP2C19, and especially CYP3A had potential DDIs with BDQ. TBI-166 did not show inhibitory effects on CYP2C8 with IC_50_ > 50 μM. The IC_50_ values of TBI-166 for CYP2C9 and CYP2C19 were 5.45 and 4.49 μM, respectively. The IC_50_ values for CYP3A4 were different with different probe substrates; those for midazolam and testosterone were 17.36 and 2.65 μM, respectively. Therefore, TBI-166 is a moderate inhibitor of the CYP3A4 pathway with an IC_50_ value of 2.65 μM (1 µM < IC_50_ < 10 µM) (Krippendorff et al., 2007).

### 3.3 Establishment and validation of HPLC-MS/MS method for pharmacokinetic study

The quantification of BDQ, M2 and TBI-166 in mice plasma was fully validated. The concentrations of BDQ, TBI-166, and M2 were measured by using HPLC-MS/MS. Supplementary Figure S1 shows representative HPLC-MS/MS chromatograms of the blank plasma (Supplementary Figure S1A), quality control plasma sample (blank plasma supplemented with BDQ 5 ng/mL, TBI-166 5 ng/mL, and M2 15 ng/mL), and IS (propranolol 100 ng/mL) (Supplementary Figure S1B). The chromatograms showed that BDQ, TBI-166, M2, and IS were completely separated by HPLC with retention times of 1.96, 1.79, 1.83, and 1.67 min, respectively. The calibration curves for the peak area ratios of BDQ/IS, TBI-166/IS, and M2/IS against BDQ (5–1,000 ng/mL), TBI-166 (5–1,000 ng/mL), and M2 (15–3,000 ng/mL) showed good linearity, with all correlation coefficients higher than 0.99. The LLOQs were 5, 5, and 15 ng/mL, for BDQ, TBI-166, and M2, respectively, and the method showed good reproducibility with precision of less than 15% for all QC samples and less than 20% for LLOQ samples. Validation results for precision, accuracy, extraction recovery, matrix effect, and stability of BDQ, M2, and TBI-166 demonstrate compliance with FDA guidelines (complete method validation results are presented in Supplementary Tables S1–S3).

### 3.4 Pharmacokinetic interaction of the combination of BDQ and TBI-166 in BALB/c mice

**FIGURE 2:**
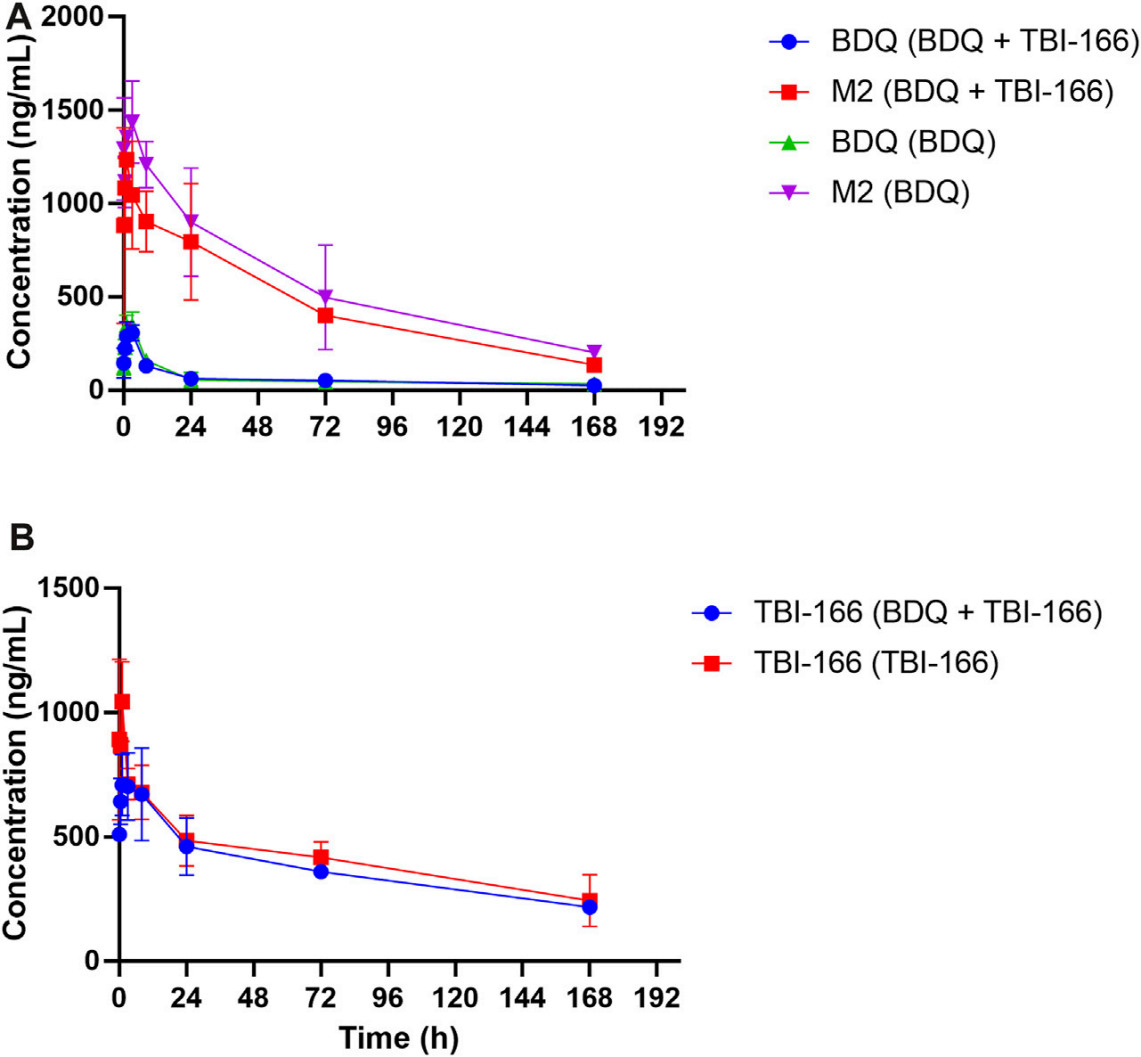
Mean plasma concentration-time profiles of **(A)** BDQ and M2 and **(B)** TBI-166 after oral administration of BDQ in BALB/c mice with or without TBI-166 (mean ± SD, *n* = 3).

Based on our *in vitro* findings, the effects of TBI-166 on the pharmacokinetic behavior of CYP3A and BDQ was investigated in BALB/c mice. Mice were treated with 20 oral doses of BDQ, TBI-166, or BDQ + TBI-166, similar to our previous research (Zhang et al., 2019), and then all mice received a final dose. The plasma concentration versus time profiles of BDQ, M2, and TBI-166 are shown in Figures 2A, B, the pharmacokinetic parameters are presented in Tables 2, 3, and the results of the lung and spleen tissue exposure are presented in Supplementary Table S4.

**TABLE 2:** Pharmacokinetic parameters of BDQ and M2 after oral administration of BDQ in mice with or without TBI-166 (mean ± SD, *n* = 3).

Parameters (unit)	BDQ (BDQ + TBI-166)		BDQ (BDQ)	M2 (BDQ + TBI-166)	M2 (BDQ)
*t*_1/2_ (h)	106.67		215.49	57.47	64.27
*T*_max_ (h)	3		3	1	3
*C*_max_ (ng/mL)	308.27 ± 24.11		349.0 ± 69.5	1,235.36 ± 22.74	1,438.31 ± 127.21
AUC_(0-last)_ (h·ng/mL)	9,971 ± 594 (101%)		9,843 ± 1,325 (100%)	76,310 ± 5,901 (66%)	115,704 ± 19,589 (100%)
AUC_(0-∞)_ (h·ng/mL)	13,842		15,695	89,814	134,430
MRT_(0-t)_ (h)	54.16		52.80	51.71	56.46

**TABLE 3:** Pharmacokinetic parameters of TBI-166 after oral administration of BDQ in mice with or without BDQ (mean ± SD, *n* = 3).

Parameters (unit)	TBI-166 (BDQ + TBI-166)	TBI-166 (TBI-166)
*t*_1/2_ (h)	132.67	142.03
*T*_max_ (h)	1	1
*C*_max_ (ng/mL)	709.45 ± 71.13	1,043.84 ± 92.53
AUC_(0-last)_ (h·ng/mL)	62,050 ± 2,519 (91%)	68,953 ± 4,843 (100%)
AUC_(0-∞)_ (h·ng/mL)	103,720	119,114
MRT_(0-t)_ (h)	65.18	66.37

**FIGURE 3:**
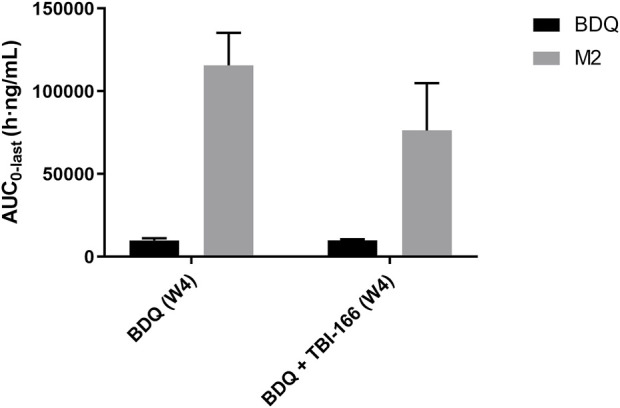
Mean AUC_0-last_ of BDQ and M2 after oral administration of BDQ in BALB/c mice with or without TBI-166 (mean ± SD, *n* = 3).

The pharmacokinetic parameters of BDQ, M2, and TBI-166 demonstrated a major DDI between BDQ and TBI-166. For both the BDQ and BDQ + TBI-166 groups, *T*_max_ of BDQ was 3 h post dose, whereas *T*_max_ of M2 was 1 h post dose in the BDQ + TBI-166 group and 3 h post dose in the BDQ group. *C*_max_ values of BDQ and M2 were similar in each group, whereas the mean M2 concentration was lower in the BDQ + TBI-166 group at 1 h post dose (85%, 1,235.36 ng/mL vs. 1,438 ng/mL) and was much lower at 3 h post dose (75%, 1,084.84 ng/mL vs. 1,438.31 ng/mL). AUC_0-last_ and MRT of BDQ were stable. In contrast, AUC_0-last_ of M2 decreased by 34%, from 115,704.59 to 76,310.39 h ng/mL are shown in Figure 3 and MRT decreased from 56.46 to 51.70 h after coadministration of BDQ with TBI-166. The M2 concentration was lower in the BDQ + TBI-166 group than in the BDQ group at each time in Figure 2A. The pharmacokinetic parameters of TBI-166 were not changed after coadministration of BDQ with TBI-166. The tissue exposure data indicated that the exposure (AUC_0-t_) for BDQ, TBI-166, and M2 was similar in the lungs and exposure increased by 1.5-fold after coadministration of BDQ with TBI-166 in the spleens.

### 3.5 Interaction of BDQ and TBI-166 in a murine model of TB

**FIGURE 4:**
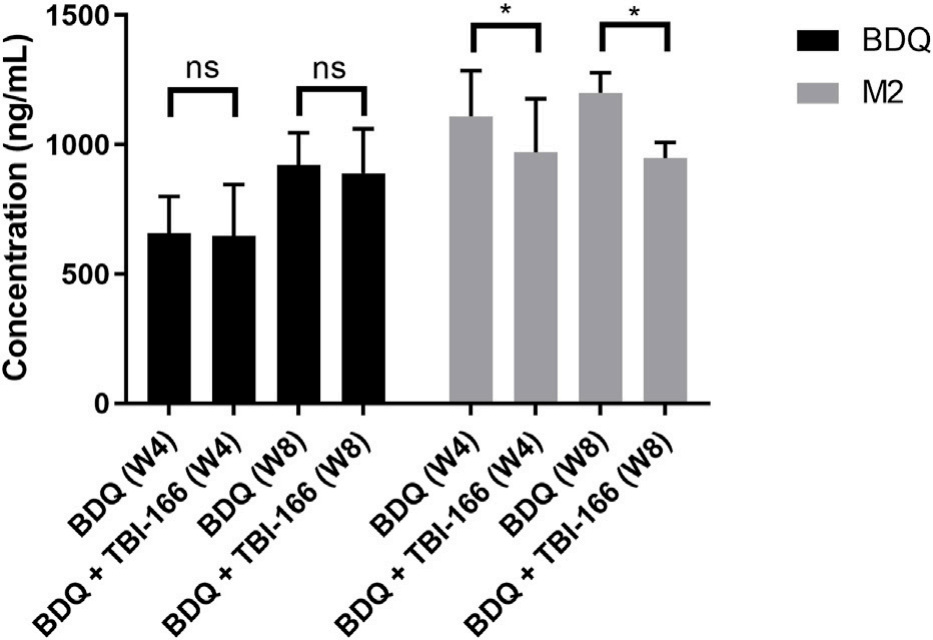
Mean plasma concentration of BDQ and M2 3 h after oral administration of BDQ in BALB/c mice infected with *Mycobacterium tuberculosis* H37Rv with or without TBI-166 (mean ± SD, *n* = 5, ns, not significant; *, *p* < 0.05).

The DDI between BDQ and TBI-166, which may affect the pharmacokinetics of BDQ in the TB-infected state, was investigated. BALB/c mice infected with *M. tuberculosis* H37Rv were treated with oral doses of BDQ, TBI-166, or BDQ + TBI-166 for 4 weeks (20 doses) or 8 weeks (40 doses), and then all mice received a final dose. The plasma concentrations of BDQ, M2, and TBI-166 3 h after the last dose are shown in Figure 4; Supplementary Table S5.

The plasma concentration of BDQ remained steady, whereas that of the toxic metabolite M2 decreased significantly from 1,109 to 970 ng/mL (87%, *p* < 0.05) after coadministration of BDQ with TBI-166 for 4 weeks. The M2 concentration was higher and decreased from 1,199 to 948 ng/mL (79%, *p* < 0.05) after coadministration of BDQ with TBI-166 for 8 weeks (Figure 4). Coadministration of BDQ had no effect on plasma concentrations of TBI-166 (Supplementary Table S5).

## 4 Discussion

We have previously reported that regimens containing the combination of BDQ and TBI-166 had stronger anti-TB activity than the HRZE first-line anti-TB regimen and the BPaL regimen, and this combination has been recommended for further study in a phase IIb clinical trial (Ding et al., 2022). Our research indicated that synergistic anti-TB effects may arise from BDQ and TBI-166 inhibiting key pathways in oxidative phosphorylation and ATP synthesis in *M. tuberculosis*, and that the combination decreased ATP content and increased the reactive oxygen species content in the bacteria significantly compared with BDQ or TBI-166 alone (Ding et al., 2022). The synergistic bactericidal activity of TBI-166 and BDQ is clear, but the potential for a DDI has not been revealed. In the present study, we systematically evaluated the DDI of BDQ and TBI-166 *in vitro* and *in vivo*.

CYPs are the main pathway by which BDQ is metabolized, and M2 is the major circulating metabolite. The involvement of CYP3A4, CYP2C8, and CYP2C19 in BDQ metabolism and M2 formation is supported by *in vitro* data, but the contributions of the CYPs had not been quantified (Liu et al., 2014). The quantitative *f*_m_ values that we obtained indicated that more than 75% of BDQ clearance is performed by the CYP3A family, and the CYP3A pathway is the main contributor to BDQ metabolism in the human liver. Because BDQ is predominantly metabolized by CYPs, we focused on assessing the possible DDI between TBI-166 and BDQ by the inhibition or inactivation of CYPs. TBI-166 inhibited the main BDQ metabolism pathway of CYP3A4 with IC_50_ values of 17.36 and 2.65 μM for probe substrates of midazolam and testosterone, respectively. A reasonable explanation for the difference in IC_50_ between the substrates is that the substrates and the inhibitor are complexed to the active site of the CYP3A4 enzyme; thus, TBI-166 inhibits CYP3A4 at a different locus with a different strength. TBI-166 is a moderate inhibitor for DDIs via the CYP3A4 pathway. The TBI-166 plasma concentration in mice treated with a human equivalent dose was lower than the IC_50_ of CYP3A4 (1.19 μM vs. 2.65 μM). This was because TBI-166 accumulation in liver tissue meant that the concentration was much higher than the IC_50_ of CYP3A4 (32.28 μg/mL, 58.18 μM) (Xu et al., 2019). The liver is the main organ in which CYPs are expressed and drug metabolism occurs, and thus the TBI-166 DDI may inhibit BDQ metabolism in the liver.

An *in vivo* pharmacokinetic study of the DDI of BDQ and TBI-166 was performed in both healthy BALB/c mice and a murine model of TB. For the BDQ + TBI-166 group, *T*_max_ of M2 decreased to 1 h post dose, which could be explained by the rapid absorption of TBI-166, reaching *C*_max_ at 1 h post dose, and the consequent inhibition of the CYP3A4 pathway. Because the CYP3A4 pathway was inhibited, the production of toxic metabolite M2 was reduced in the BDQ + TBI-166 group compared with the BDQ group. The systemic exposure to M2 was decreased by coadministration of BDQ and TBI-166. Pharmacokinetic parameters, such as *t*_1/2_, *C*_max_, MRT, and AUC_(0–t)_, of M2 were decreased in the BDQ + TBI-166 group. The AUC_0-last_ of M2 decreased to 66% of that of the BDQ group. The break point of a potential DDI is an area under the curve ratio (AUCR) of 0.8–1.2 (Sudsakorn et al., 2020) or based on the standard bio-equivalence criteria (80%–125%). The AUCR of M2 in the BDQ + TBI-166 group was 0.66 (66%), indicating a potential DDI between BDQ and TBI-166.

Our interaction study in a murine model of TB confirmed that the interaction between BDQ and TBI-166 could reduce the C_max_ of the toxic metabolite M2, may resulting in lower M2 exposure. Furthermore, we observed that this reduction in M2 exposure increased from 4 weeks to 8 weeks of treatment. Treatment for MDR-TB takes 9–24 months, and thus the large decrease in the concentration of M2 during treatment is important. CYP3A4 has been reported to be the isoform most affected in the downregulation of inflammation-related CYPs, which may lead to lower metabolism, longer half-life, and increased plasma concentration of the CYP3A4 substrate drugs used for TB treatment (Lenoir et al., 2021). This may explain why the concentration of BDQ was significantly lower in healthy mice than in the murine model of TB.

The results of the *in vitro* metabolism experiments and *in vivo* preclinical pharmacokinetic data indicated that coadministration of BDQ and TBI-166 resulted in a DDI via the CYP3A4 pathway, which inhibited BDQ metabolism to M2, reducing exposure. Compared with BDQ, M2 has 3- to 6-fold lower antimycobacterial activity but 50 times higher cardiotoxicity by inducing phospholipidosis. Clinical research has indicated that M2 is the main contributor to drug-induced QT prolongation related to BDQ (Tanneau et al., 2022). M2 is further n-demethylated to N-didesmethyl metabolite (M3). It is likely that the same enzymes involved in clearance of M2 as of its formation (Svensson et al., 2013). Reducing exposure to M2 will decrease the rate of adverse cardiovascular events associated with clinical BDQ treatment, which will improve safety and tolerability for MDR-TB patients. Our results indicated that M2 is almost entirely produced from BDQ by the CYP3A4 pathway. Clinical research has also shown that coadministration of CYP inhibitors with BDQ has a synergistic effect and reduces the ratio of BDQ converted to M2 (Brill et al., 2017). Coadministration of BDQ and TBI-166 resulted in the same exposure of both BDQ and TBI-166 for all treatments. The pharmacokinetics results indicated that the synergistic anti-TB effects of the combination may arise from BDQ and TBI-166 co-inhibiting key pathways in *M. tuberculosis* rather than the DDI of this combination. These results highlight that properly designed clinical trials and therapy are required for regimens containing a combination of BDQ and TBI-166. The pharmacokinetic DDI mechanism of TBI-166 consists of the pharmacodynamic interactions of the synergistic bactericidal activity with BDQ and the reduced cardiotoxicity associated with toxic metabolites.

However, there are several limitations to the current study. First, the sampling data from the pharmacokinetics research in BALB/c mice were sparse and the concentrations at each time were only taken from three mice; therefore, no statistical analysis of systemic exposure (AUC) was possible. Thus, although the AUCR of the decrease in M2 showed that there is a meaningful DDI, statistically significant results will clarify the relative M2 decrease with the coadministration of BDQ and TBI-166. Another limitation is that we used uninfected mice for the pharmacokinetic studies and we validated the existence of the DDI in the BALB/c mouse TB model by the change in *C*_max_ upon coadministration of BDQ and TBI-166. Although a reduction in M2 was observed with coadministration of BDQ and TBI-166, the change in QT prolongation was not evaluated due to model limitations. Furthermore, we do not yet understand why when the exposure of M2 was decreased, its parent drug BDQ in both the plasma and target organ had the same exposure in all treatments. It is possible that alternative pathways were activated that maintain clearance of BDQ after TBI-166 inhibits CYP3A4, other pathways compensate for the increased clearance of BDQ and potentially generate fewer toxic metabolite M2. A future mass balance study may provide an answer.

## 5 Conclusion

We confirmed a potential DDI between BDQ and TBI-166 through *in vitro* and *in vivo* studies. Our findings demonstrated that coadministration of BDQ and TBI-166 may significantly reduce exposure to toxic metabolite M2 by inhibiting the CYP3A4 pathway, which could improve the safety and efficacy of clinical TB treatment and provide a foundation for an oral short course regimen for MDR-TB and XDR-TB treatment of coadministration of BDQ and TBI-166. The benefits of the combination of BDQ and TBI-166 are worth evaluating in a phase IIb clinical trial.

## Data Availability

The original contributions presented in the study are included in the article/Supplementary Material, further inquiries can be directed to the corresponding authors.
