# Supplementary material for: Coadministration of bedaquiline and pyrifazimine reduce exposure to toxic metabolite N-desmethyl bedaquiline
Source: Front Pharmacol. 2023 Oct 4;14:1154780. doi: 10.3389/fphar.2023.1154780 (PMC10582325; doi:10.3389/fphar.2023.1154780)
Supplement: Supplementary file 1 [file Table1.DOCX]

Supplementary Material

Article Title

**Yangming Ding^1^, Haiting Liu^1^, Furun Wang^2^, Lei Fu^1^, Hui Zhu^1^, Shuang Fu^2^, Ning Wang^1^,** **Xiaomei Zhuang^2*#^, Yu Lu ^1*#^**

*** Correspondence:**

Yu Lu
luyu4876@hotmail.com

Xiaomei Zhuang
xiaomeizhuang@163.com

# Yu Lu and Xiaomei Zhuang contributed equally to this work

# Supplementary Figures and Tables

Table S1 Precision and accuracy of LC-MS/MS method for the determination of BDQ, M2 and TBI-166 in mice plasma(mean ± SD, n=6)

| Analytes | Concentration  (ng/mL) | Intra-day precision (n=6) | | |  | Inter-day precision (n=18) | | |  |
| --- | --- | --- | --- | --- | --- | --- | --- | --- | --- |
|  |  | Determined  (Mean ± SD, n=6, ng/mL) | RE  (%) | CV  (%) |  | Determined  (Mean ± SD, n=18, ng/mL) | RE  (%) | CV  (%) | |
| BDQ | 5 | 5.21 ±0.11 | 4.20 | 2.03 |  | 5.35 ±0.52 | 7.00 | 9.66 | |
|  | 15 | 15.46±0.43 | 3.07 | 2.77 |  | 15.99±1.48 | 6.60 | 9.28 | |
|  | 75 | 74.03 ±3.02 | -1.29 | 4.08 |  | 77.00 ±5.05 | 2.67 | 6.56 | |
|  | 750 | 694.83±20.05 | -7.36 | 2.89 |  | 700.82±23.63 | -6.56 | 3.37 | |
| M2 | 15 | 14.58±0.31 | -2.80 | 2.10 |  | 14.74±0.86 | -1.73 | 5.81 | |
|  | 45 | 48.26±1.01 | 7.24 | 2.10 |  | 50.68±4.44 | 12.62 | 8.76 | |
|  | 225 | 235.00±14.04 | 4.44 | 5.98 |  | 243.94±17.21 | 8.42 | 7.05 | |
|  | 2250 | 1974.67±35.45 | -12.24 | 1.80 |  | 2008.78±97.90 | -10.72 | 4.87 | |
| TBI-166 | 5 | 5.32 ±0.10 | 6.40 | 1.94 |  | 5.23 ±0.45 | 4.60 | 8.55 | |
|  | 15 | 16.23±1.87 | 8.20 | 11.51 |  | 14.94±1.97 | -0.40 | 13.22 | |
|  | 75 | 72.62±3.71 | -3.17 | 5.11 |  | 74.43±4.20 | -0.76 | 5.64 | |
|  | 750 | 777.67±25.03 | 3.69 | 3.22 |  | 763.63±33.28 | 1.82 | 4.36 | |

Table S2 Extraction recovery and matrix effect of BDQ, M2 and TBI-166 in mice plasma (mean ± SD, n=6)

| Analytes | Concentration  (ng/mL) | Extraction recoveries  (%) | RSD  (%) | Matrix effect (%) | RSD (%) |
| --- | --- | --- | --- | --- | --- |
| BDQ | 15 | 98.58±5.37 | 5.44 | 88.84±4.19 | 4.71 |
|  | 75 | 100.40±7.04 | 7.01 |  |  |
|  | 750 | 108.03±5.01 | 4.63 | 98.96±7.74 | 7.82 |
| M2 | 45 | 108.41±4.79 | 4.41 | 77.35±4.04 | 5.23 |
|  | 225 | 104.24±6.68 | 6.41 |  |  |
|  | 2250 | 113.51±5.31 | 4.67 | 93.16±5.87 | 6.30 |
| TBI-166 | 15 | 100.66±3.70 | 3.67 | 86.81±7.59 | 8.74 |
|  | 75 | 100.06±12.25 | 12.24 |  |  |
|  | 750 | 105.44 ±6.21 | 5.87 | 94.39 ±4.56 | 4.82 |

Table S3 Stability of BDQ, M2 and TBI-166 in mice plasma (mean ± SD, n=3)

| Analytes | Concentration  (ng/mL) | Room temperature for 24 h | |  | 4℃ for 24 h | |  | Freeze-thaw three cycles | |  |  |
| --- | --- | --- | --- | --- | --- | --- | --- | --- | --- | --- | --- |
|  |  | RE  (%) | RSD  (%) |  | RE  (%) | RSD  (%) |  | RE  (%) | RSD  (%) |  |  |
| BDQ | 15 | -12.13 | 2.80 |  | -13.87 | 4.13 |  | -6.80 | 11.52 |  |  |
|  | 75 | 3.24 | 9.73 |  | -11.67 | 6.00 |  | 1.31 | 4.23 |  |  |
|  | 750 | -5.54 | 7.11 |  | -6.15 | 2.64 |  | -10.24 | 3.01 |  |  |
| M2 | 45 | 9.38 | 7.98 |  | 5.42 | 12.41 |  | 12.58 | 10.66 |  |  |
|  | 225 | 17.30 | 4.77 |  | 13.08 | 5.94 |  | 17.75 | 11.11 |  |  |
|  | 2250 | 4.03 | 12.00 |  | 9.97 | 3.41 |  | 6.90 | 2.90 |  |  |
| TBI-166 | 15 | -6.20 | 13.12 |  | -15.07 | 17.25 |  | -14.27 | 16.87 |  |  |
|  | 75 | -6.81 | 12.08 |  | 1.87 | 5.76 |  | -5.39 | 5.11 |  |  |
|  | 750 | -10.81 | 9.20 |  | -12.65 | 1.07 |  | -13.50 | 2.95 |  |  |

| Analytes | Concentration  (ng/mL) | -40℃ for 7 d | |  | In auto-sample for 24 h | |  |  |  |
| --- | --- | --- | --- | --- | --- | --- | --- | --- | --- |
|  |  | RE  (%) | RSD  (%) |  | RE  (%) | RSD  (%) |  |  |  |
| BDQ | 15 | -10.20 | 0.80 |  | -12.60 | 16.56 |  |  |  |
|  | 75 | -11.09 | 11.88 |  | 5.89 | 9.41 |  |  |  |
|  | 750 | -6.34 | 1.70 |  | -2.46 | 10.50 |  |  |  |
| M2 | 45 | 15.73 | 7.55 |  | 14.04 | 1.65 |  |  |  |
|  | 225 | 2.77 | 10.38 |  | 17.58 | 1.45 |  |  |  |
|  | 2250 | 9.33 | 2.34 |  | 4.83 | 14.79 |  |  |  |
| TBI-166 | 15 | -2.20 | 13.74 |  | 18.87 | 10.10 |  |  |  |
|  | 75 | -1.01 | 9.03 |  | -12.61 | 8.14 |  |  |  |
|  | 750 | -13.53 | 2.20 |  | -7.77 | 7.39 |  |  |  |

Table S4 AUC_(0-t)_ and tissue-to-plasma ratio (*K*_p_) of BDQ and M2 after oral administration of BDQ in mice that were combined with or without TBI-166 (Mean±SD, n=3)

| Groups | AUC_(0-t)_ | *K*_p_ | |  | AUC_(0-t)_  h·μg/g or h·μg/mL | *K*_p_ |
| --- | --- | --- | --- | --- | --- | --- |
|  | h·μg/g or h·μg/mL |  |  |  |  |  |
| BDQ  (BDQ + TBI-166) |  | |  | BDQ  (BDQ) |  |  |
| Plasma | 9.97 ± 0.79 | |  |  | 10.15 ± 1.33 |  |
| Lung | 200.79 ± 19.63 | | 20.14 |  | 200.17 ± 25.47 | 19.72 |
| Spleen | 202.13 ± 27.37 | | 20.27 |  | 132.73 ± 10.99 | 13.07 |
|  |  | |  |  |  |  |
| M2  (BDQ + TBI-166) |  | |  | M2  (BDQ) |  |  |
| Plasma | 76.25 ± 8.10 | |  |  | 115.73 ± 25.44 |  |
| Lung | 4940.02 ± 756.58 | | 64.79 |  | 4827.39 ± 818.42 | 41.72 |
| Spleen | 6358.44 ± 1054.47 | | 83.39 |  | 4703.39 ± 1052.83 | 40.65 |
|  |  | |  |  |  |  |
| TBI-166  (BDQ + TBI-166) |  | |  | TBI-166  (TBI-166) |  |  |
| Plasma | 62.05 ± 2.52 | |  |  | 68.95±4.84 |  |
| Lung | 321.07±52.91 | | 5.17 |  | 378.66±104.67 | 5.49 |
| Spleen | 483.54 ± 94.75 | | 7.79 |  | 296.09±27.68 | 4.29 |

Table S5 Mean plasma concentration of BDQ, M2 and TBI-166 at three hours after oral administration of BDQ in mice that were combined with or without TBI-166 (Mean ± SD, n=5)

| Group  (WEEK) | BDQ  (ng/ml) | M2  (ng/ml) | TBI-166  (ng/ml) |
| --- | --- | --- | --- |
| BDQ （W4） | 656.59±141.64 | 1109.12±176.23 | <LLOQ |
| TBI-166 （W4） | <LLOQ | <LLOQ | 1375.79±123.54 |
| BDQ + TBI-166（W4） | 647.02±199.43 | 969.68±206.81 | 1383.02±411.59 |
|  |  |  |  |
| BDQ （W8） | 919.86±126.03 | 1199.24±79.33 | <LLOQ |
| TBI-166 （W8） | <LLOQ | <LLOQ | 1323.76±311.89 |
| BDQ + TBI-166 （W8） | 889.43±171.00 | 948.31±60.21 | 1330.40±133.72 |


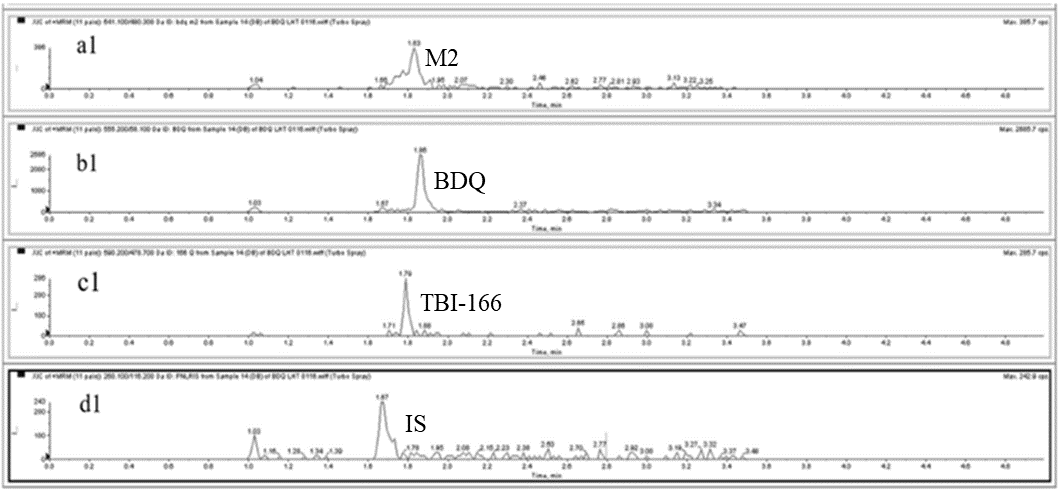


A


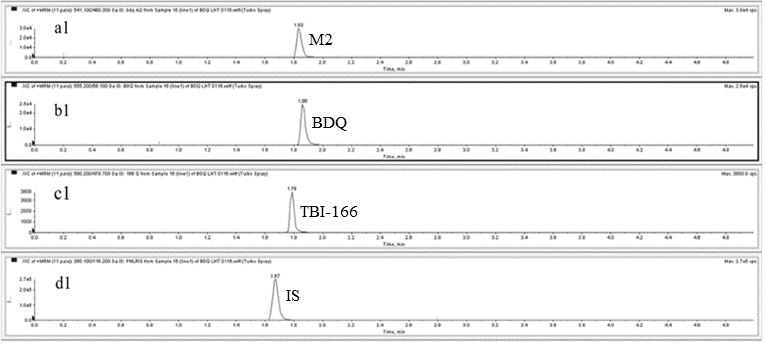


B

FIGURE S1. LC-MS/MS chromatograms of (A) blank plasma (B) blank plasma spiked with BDQ(5 ng/ml), TBI-166(5 ng/ml), BDQ-M2(15 ng/ml), and propranolol as the IS (100 ng/ml)

a1: M2, b1: BDQ, c1: TBI-166 d1: IS.
